# A Retrospective Study of Coronary Artery Bypass Grafting with Low-Thermal Plasma Dissection Device Compared to Conventional Monopolar Electrosurgery

**DOI:** 10.21470/1678-9741-2020-0386

**Published:** 2021

**Authors:** Dincer Uysal, Erdogan Ibrisim

**Affiliations:** 1 Department of Cardiovascular Surgery, Suleyman Demirel Universitesi Tip Fakultesi, Isparta, Turkey.

**Keywords:** Coronary Artery Bypass, Electrosurgery, Pain, Postoperative, Visual Analog Scale

## Abstract

**Introduction:**

The objective of this study is to compare the effects of conventional monopolar electrosurgery (CMES) and low-thermal plasma kinetic cautery (PKC) on complications such as bleeding, abnormal wound healing, pain, and drainage in patients who underwent on-pump coronary artery bypass grafting (CABG).

**Methods:**

This retrospective clinical study included 258 patients undergoing CABG; the patients were randomized to PKC (PEAK PlasmaBlade, n=153) and CMES (n=105) groups. The patients’ clinical data were examined retrospectively for biochemical variables, postoperative drainage, post-surgery erythrocyte suspension transfusion count, surgical site pain examined with visual analogue scale (VAS), and wound healing. Two-sided *P*-value > 0.05 was considered as statistically significant.

**Results:**

The median post-surgery erythrocyte suspension transfusion number was significantly lower with PKC compared to CMES (0 [0-1] *vs*. 1 [1-4], respectively, *P*<0.001). Mean postoperative drain output and time until removal of drain tubes were significantly lower with PKC compared to CMES (300±113 *vs*. 547±192 and 1.95±1.5 *vs*. 2.44±1.8; *P*<0.001 and *P*=0.025, respectively). Mean VAS score for spontaneous and cough-induced pain were significantly lower with PKC compared to CMES (1.98±1.51 *vs*. 3.94± 2.09 and 3.76±1.46 *vs*. 5.6±1.92; *P*<0.001 for both comparisons). Reoperation due to bleeding was significantly higher with CMES compared to PKC (0 *vs*. 11 [7.2%], *P*=0.001).

**Conclusion:**

Use of PKC during CABG considerably reduces postoperative drainage, need for blood transfusion, reoperation due to bleeding, and postoperative pain. PCK appears to be a good alternative to CMES for CABG.

**Table:** 

Abbreviations, acronyms & symbols				
**AST** **BUN** **CABG** **CI** **CMES** **COLD** **CPB** **CRP** **EF**	**= Aspartate aminotransferase** **= Blood urea nitrogen** **= Coronary artery bypass grafting** **= Confidence interval** **= Conventional monopolar electrosurgery** **= Chronic obstructive lung disease** **= Cardiopulmonary bypass** **= C-reactive protein** **= Ejection fraction**		**IHD** **LIMA** **MSV** **PKC** **SD** **USA** **VAS** **WBC**	**= Ischemic heart disease** **= Left internal mammary artery** **= Major saphenous vein** **= Plasma kinetic cautery** **= Standard deviation** **= United States of America** **= Visual analogue scale** **= White blood cell**

## INTRODUCTION

Ischemic heart disease patients undergoing coronary artery bypass grafting (CABG) have a significantly increased risk of complications such as surgical site infection, abnormal wound healing, and blood loss (complication rates are 3%, 19%, and 10%, respectively) suggesting that major comorbidities such as old age, diabetes, low cardiac output, vascular circulation problems, and use of anticoagulant drugs may compromise outcomes ^[1,2]^. However, surgical technique-related and modifiable factors can minimize these complications. One of them is the choice of device used for cutting tissues and ablation of blood vessels. The standard and commonly used device is the conventional monopolar electrosurgery (CMES) device, which uses continuous-waveform radiofrequency energy delivered by an uninsulated metal electrode to cut tissue by thermal ablation, producing simultaneous hemostasis ^[3]^. CMES device provides an operating temperature in the range of 250 ºC to 350 ºC ^[3]^. This high temperature results in significant thermal damage, widespread deep tissue necrosis of the incised tissues, and delayed wound healing, and has a potential to injure adjacent structures such as the heart, lung, vagus and phrenic nerves, and grafts such as internal mammary artery or vena saphena magna.

As an alternative to CMES cautery, plasma kinetic coagulation and resection uses radiofrequency energy by utilizing bipolar plasma kinetic technology. Plasma kinetic cautery (PKC) (PEAK PlasmaBlade®, Medtronic Advanced Energy LLC, Portsmouth, United States of America [USA]) is a novel, low-thermal-injury electrosurgical instrument that uses very brief (40 µs) high-frequency pulses of radiofrequency energy to induce electrical plasma hemostasis along the edge of a thin (approximately 12.5 µm), flat, 99.5% insulated electrode ^[3]^. PKC produces an operating temperature between 40 ºC and 100 ºC with its electrode ^[3]^. The efficacy and reliability of PKC has been shown in human ophthalmologic dissection (retina and other intraocular tissues), bariatric surgery, mastectomy, and urologic surgery where PKC was reported to have similar precision as a scalpel and hemostatic control to CMES ^[3-9]^.

In recent years, PKC hemostasis and incision has started to be used in a small number of cardiac surgery clinics. However, no study on the efficiency and reliability of PKC in patients undergoing CABG is currently available in the literature.

In this retrospective clinical study, we compared the effects of CMES and PKC on complications such as bleeding, abnormal wound healing, surgical site pain, and drainage in patients who underwent on-pump CABG.

## METHODS

### Patient Selection

This is a retrospective observational study that was carried out in accordance with the principles outlined in the Declaration of Helsinki. Ethical approval was obtained from the local ethical committee (13.05.2020/143) and all patients provided written informed consent for using of their medical records prior to the planned surgery. Two hundred fifty-eight adult ischemic heart disease patients who underwent on-pump CABG between January 2016 to December 2019 were included in the study. The inclusion criteria were: patients aged between 18 and 80 years and who underwent CABG requiring cardiopulmonary bypass (CPB). Exclusion criteria were: low ejection fraction (EF < 30%), emergency operation, left ventricular aneurysm, reoperation, concomitant procedures (such as valve replacement, carotid endarterectomy, left ventricular aneurysm resection, and others), perioperative myocardial infarct, preoperative diagnosis of chronic renal or hepatic insufficiency, malignancy, systemic inflammatory disease, history of cardiac surgery, lack of consent, and failure to obtain all the patient data necessary for the study.

The patients who underwent CABG with PKC only (n=153) were assigned as the study group while patients who underwent surgery with CMES and blade (n=105) were assigned as the control group. Low-density lipoprotein cholesterol (> 160 mg/dL [4.1 mmol/L]), non-high-density lipoprotein cholesterol (> 190 mg/dL [4.9 mmol/L]), and triglycerides (> 150 mg/dL, nonfasting) were used to determine the presence of hyperlipidemia, and hypertension is defined as a systolic blood pressure ≥ 140 mm Hg or a diastolic blood pressure of ≥ 90 mm Hg.

### Biochemical Parameters

Preoperative and postoperative hematologic and biochemical parameters were collected from the patients’ records. Complete blood counts were analyzed with an automatic 24-parameter blood count device (Bayer Pentra 80-Siemens Healthcare Diagnostics Products, Marburg, Germany). All the other biochemical parameters were analyzed using fresh plasma with a Siemens BCS measuring device (Siemens Healthcare Diagnostics Products GmbH 2008 Marburg, Germany).

### Histopathologic Procedure

All harvested arterial segments were immersed in 10% neutral formalin solution and embedded in paraffin. Sections were cut with a cryostat at 6-µm thickness and were prepared for histochemical studies. These sections were evaluated for luminal endothelial integrity in the left internal mammary artery (LIMA) and in the vaso vasorum.

Also, to represent the cutting edge burnt tissue, thymus tissue was totally excised following median sternotomy in a patient and the thymus tissue was incised full section with both cautery systems, then the cutting edge photographs were taken.

### Visual Analogue Scale Pain Score

Visual analogue scale (VAS) pain scores were obtained from the patients’ records. The intensity of pain was evaluated after extubation every six hours, for a total of 72 hours (12 times), during the intensive care unit stay and whenever the patient had complaints and need for analgesics. Patients were asked to state their pain intensity during rest and while coughing using VAS. Pain intensity was measured by critical care nurses in a routine workup who were blinded to the protocols and results of the study but were fully trained and familiar with VAS.

### Anesthetic Protocol

Patients were premedicated with 0.05 mg/kg of intramuscular midazolam (Dormicum, Roche Pharmaceuticals, Nutley, New Jersey, USA) 30 minutes before surgery. Intubation was carried out after the induction of anesthesia with 2% lidocaine (Aritmal, Osel Ilaç San. ve Tic. A.S. Istanbul, Turkey), 0.05 mg/kg of midazolam, 25-30 mcg/kg of fentanyl citrate (Abbojet, Abbott Laboratories, Abbott Park, Los Angeles, USA), 1 mg/kg of ketamine (Ketalar, Zentiva Saglık Urünleri San. ve Tic. A.S., Kırklareli, Turkey), 0.2 mg/kg of etomidate (Lipuro, B. Braun Melsungen AG, Berlin, Germany), and 0.1 mg/kg of pancuronium (Pavulon; Santa Farma, United Arab Emirates). A nasoesophageal temperature probe and capnograph were placed, following the insertion of a nasogastric tube and Foley urine catheter. Before the commencement of CPB, 300 IU/kg of systemic heparin was administered first, and then additional heparin doses were administered to keep activated clotting time (Hemochron 801, ITC, USA) > 480 seconds. The process was reversed by the administration of protamine hydrochloride (Protamin ICN, Mefar A.S., Istanbul, Turkey) at the end of the surgery. Anesthesia was maintained with 0.05 mg/kg/min of propofol (Diprivan, AstraZeneca Türkiye İlaç Sanayi ve Ticaret Ltd. Şti., Istanbul, Turkey) and 25 mcg/kg/min of remifentanyl (Ultiva, GlaxoSmithKline Manufacturing S.p.A, Milan, Italy) infusion, each for two hours, with the administration of 2 mg of pancuronium. Dopamine (5 µg/kg/min) and dobutamine (5 µg/kg/min) were used to facilitate weaning off CPB, as necessary. No patient in either group required high dose of inotrope support post-CPB or postoperatively. All subjects received methylprednisolone (1-2 mg/kg) and cefazolin sodium (1000 mg) prior to skin incision. None received intraoperative steroids.

### Surgical Protocol

All operations were performed by the same surgical team. During surgery, two different cautery systems were used. In the study group, only PKC was used for full-thickness skin incisions, bleeding hemostasis, and preparation of LIMA, which was prepared as a pedicled graft and major saphenous vein (MSV) grafts. For the application of PKC, an ultrasonic scalpel (PEAK PlasmaBlade, Medtronic, Portsmouth, USA) was used. A radiofrequency power source (PULSAR II Generator, Medtronic, Portsmouth, USA) of 160 W at a radiofrequency of 340-450 Hz was used for the supply of electrosurgery and PK3 mode at 340 V2 was applied for tissue resection. The incident power was 160 W during cutting and 80 W for coagulation. In the control group, routine surgery was performed with blade and CMES. For CMES, a monopolar reusable cautery pen (Denizler Med İstanbul, Turkey) was used with Petkot 500S 220V AC 50-Hz energy input (Petas, Ankara, Turkey) for electrosurgical energy. Monopolar mode at 80-110 W was used for cutting. The incident power was 70 W for coagulation and 40 W for tissue cutting.

### Cardiopulmonary Bypass Technique

Following median sternotomy, LIMA and MSV grafts were prepared for all patients. After systemic heparin administration, the artery was clipped, wrapped in a papaverine-soaked cloth, and stored under the manubrium sterni until anastomosis. A redundant 1-cm long arterial segment proximal to the clip was cut off and preserved in formalin for laboratory examination. After this, an aorto-two stage cannulation CPB was conducted and an antegrade cardioplegia cannula was placed on the ascending aorta. A roller pump (Jostra HL20, Herrlingen, Germany) was used in all cases. The CPB of each patient consisted of a cardiotomy reservoir, a tubing set, a hollow fiber membrane oxygenator, and a 40 µm arterial line filter. Mild hypothermia was applied with a nasopharyngeal temperature of 32 ºC and a non-pulsatile flow of 2.4 l/min/m^2^ and a mean arterial pressure of 55-65 mmHg was maintained during the CPB. Hemodilution was achieved with a hematocrit level of 26%. The circuits were primed with a mixture of 500 ml of succinyl gelatine 6 % (Gelofusine IV, B. Braun Medikal Dış Ticaret A.Ş, Esenler, Istanbul, Turkey), 1000 ml of isolyte, 100 ml of mannitol 20 %, NaHCO_3_ 8.4%, 250 mg of methylprednisolone (Prednol 250, Mustafa Nevzat Inc., Istanbul, Turkey), and 1 g of cefazolin (Cefamezin 1 g, Zentiva Saglık Urünleri San. ve Tic. A.S., Istanbul, Turkey) at room temperature (20 ºC) adding up to a total of 1600 ml fluid. Myocardial protection was provided via intermittent (20 min intervals) antegrade cold blood cardioplegia in a standard formula. The cardioplegic solution was initiated at a volume of 1000 ml and repeated in volumes of 500 ml every 20 minutes, reaching a total of 2000-2500 ml, depending on the patient. Patients were weaned off the CPB systematically after completing all vascular anastomosis and ensuring that the entire surgical area was free of any bleeding site. At least two chest tubes (one for mediastina and one for left pleural cavity) were placed in all patients.

Erythrocyte suspension and fresh frozen plasma were given at the anesthesiologist's discretion. Blood transfusion was performed only if the hemoglobin level was < 7 mg/dl, hematocrit value was < 25 %, and if any clinical sign of blood loss existed.

### Data Collection

Preoperative and postoperative levels of hemoglobin, hematocrit, neutrophils, platelets, blood urea nitrogen (BUN), creatinine, alanine aminotransferase, C-reactive protein (CRP), and EF (%) were obtained from the electronic database of the hospitals’ patient file system. Clinical patient data were collected by reviewing medical and nursing records. Several parameters were determined such as age, body mass index, blood pressure, operative time points, time until drain removal, total volume of drain output, postoperative VAS pain scores, complications, blood transfusion, revision for bleeding, and mortality. Abnormal wound healing included wound dehiscence, sternal dehiscence, surgical site infection, and deep tissue necrosis.

### Statistics

Data analyses were carried out using IBM Corp. Released 2017, IBM SPSS Statistics for Windows, Version 25.0, Armonk, NY: IBM Corp. Results were expressed as mean and standard deviation or number and percent. At first, all data were examined by Shapiro-Wilk test to determine whether intraindividual differences were distributed normally. As a normal Gaussian distribution was not found, comparisons of differences between groups were carried out using Mann-Whitney U test for non-parametric data, and χ^2^-test or Fisher’s exact test for categorical variables. Data for different time points were compared with related-samples Wilcoxon signed-rank test. Two-sided *P*-value > 0.05 was considered as statistically significant.

## RESULTS

Demographic variables are shown in Table 1, which indicates that the study and control groups were comparable for the presence of risk factors (age, gender, body surface area, systolic/diastolic blood pressure, hypertension, smoking, insulin-dependent diabetes mellitus, peripheral vascular disease, chronic obstructive lung disease, family history of ischemic heart disease, and EF [%]). There were no statistically significant differences in any of the demographic parameters between the groups except the prevalence of hyperlipidemia. The PKC group showed a significantly higher prevalence of hyperlipidemia compared to the CMES group (*P*=0.01).

**Table 1:** Patients' preoperative demographic variables.

	Groups		*P* -value
	Conventional monopolar electrosurgery (CMES) (n=105)	Plasma kinetic cautery (PKC) (n=153)	
Age (years)± SD	63±9	64±9	0.472
Sex, female, n (%)	39 (37.1)	64 (41.8)	0.266
Body surface area (kg/m^2^)	1.85±0.21	1.86±0.18	0.847
Systolic blood pressure (mmHg)	128±18	130±22	0.655
Diastolic blood pressure (mmHg)	68±11	68±12	0.926
Hypertension, n (%)	74 (70.5)	105 (68.6)	0.43
Smoking, n (%)	62 (59)	75 (49)	0.072
Diabetes mellitus, n (%)	38 (36.2)	57 (37.3)	0.484
Hyperlipidemia, n (%)	63 (60)	68 (44.4)	0.01
Peripheral vascular disease, n (%)	12 (11.4)	16 (10.5)	0.479
COLD, n (%)	26 (24.8)	29 (19)	0.167
Family history of IHD, n (%)	15 (14.3)	18 (11.8)	0.34
Preoperative anticoagulation	63 (60.6)	76 (49.7)	0.23
EF (%)	55±9	54±10	0.248
Hemoglobin (mg/dl), (mean±SD)	13.86±1.87	13.55±1.81	0.179
Hematocrit %, (mean±SD)	40.48±5.12	39.97±4.97	0.434
Activated partial thromboplastin time (sn), (mean±SD)	34.92±2.88	34.92±2.86	0.488
International Normalized Ratio (mean±SD)	0.95±0.08	0.93±0.084	0.358

Statistical evaluation of the differences between the groups was carried out with Mann-Whitney U test for non-parametric data and X^2^-test or Fisher's exact test for categorical variables

*P -value is for testing the null hypothesis that the CMES group values (means or proportions) are equal to the ones from the PKC group. All tests are two-sided COLD=chronic obstructive lung disease; EF=ejection fraction; IHD=ischemic heart disease; SD=standard deviation

Both CMES and PKC groups showed comparable mean surgery time (296±65 *vs*. 299±71, respectively, *P*=0.711), duration of CPB (75.42±28.96 *vs*. 80.45±24.68, respectively, *P*=0.137), cross-clamping time (45.75±16.16 *vs*. 48.55±16.48, respectively, *P*=0.123), in-patient stay (9±3 *vs*. 9±3, respectively, *P*=0.66), and median number of bypass (3 [1-5] *vs*. 3 [1-5], *P*=0.852, respectively) (Table 2). The median erythrocyte suspension transfusion number (0 [0-1] *vs*. 1 [1-4]) was significantly lower in the study (PKC) group after surgery compared to the control (CMES) group (*P*<0.001) (Table 2). The mean postoperative drain output (300±113 *vs*. 547±192, *P*<0.001) and time until drain tubes removal (1.95±1.5 *vs*. 2.44±1.8, *P*=0.025) were also significantly lower in the study group.

**Table 2:** Intraoperative and postoperative data of both dissection techniques.

	Groups		*P* -value
	Plasma kinetic cautery (PKC) (n=153)	Conventional monopolar electrosurgery (CMES) (n=105)	
Total surgery time (min±SD)	296±65	299±71	0.711
Total CPB time (min±SD)	75.42±28.96	80.45±24.68	0.137
Total cross-clamping time (min±SD)	45.75±16.16	48.55±16.48	0.123
In-patient stay (days±SD)	9±3	9±3	0.66
Numbers of bypass, median (min-max)	3 (1-5)	3 (1-5)	0.852
Erythrocyte suspension transfusion, median (min-max)	0 (0-1)	1 (1-4)	< 0.001
Postoperative drain output (ml±SD)	300±113	547±192	< 0.001
Time until drain tubes removal (days±SD)	1.95±1.5	2.44±1.8	0.025

Comparison of intraoperative and postoperative parameters with the use of low-thermal plasma dissection device (PKC) and monopolar electrosurgery (CMES). Statistical evaluation of the differences between the groups was carried out with Mann-Whitney U test for non-parametric data, and X^2^-test or Fisher's exact test for categorical variables

*P -value is for testing the null hypothesis that CMES group values (means or proportions) are equal to the ones from the PKC group. All tests are two-sided CPB=cardiopulmonary bypass; SD=standard deviation

Mean VAS score for spontaneous (1.98 ±1.51 *vs*. 3.94± 2.09, *P*<0.001) and cough-induced (3.76±1.46 *vs*. 5.6±1.92, *P*<0.001) pain were significantly lower in the study group compared to the control group (Table 3). Reoperation due to bleeding was significantly high in the control group (0 *vs*. 11 [7.2 %], *P*=0.001) (Table 3). The incidence of postoperative complications such as thromboembolic event, atrial fibrillation, sternal and leg incision complication, and mortality were statistically similar in both groups (Table 3).

**Table 3:** Postoperative data of complications and VAS score for both groups.

	Groups		*P* -value
	Plasma kinetic cautery (PKC) (n=153)	Conventional monopolar electrosurgery (CMES) (n=105)	
VAS score for spontaneous pain (mean±SD)	1.98 ±1.51	3.94± 2.09	<0.001
VAS score for cough-induced pain (mean±SD)	3.76±1.46	5.6±1.92	<0.001
Sternal incision complication, n (%)	2 (1.9)	6 (3.9)	0.298
Leg incision complication, n (%)	3 (2.9)	6 (3.9)	0.464
Postoperative bleeding revision, n (%)	0	11 (7.2)	0.003
Thromboembolic event, n (%)	1 (0.9)	4 (2.6)	0.122
Atrial fibrillation, n (%)	32 (30.5)	33 (21.6)	0.71
Mortality, n (%)	2 (1.9)	6 (3.9)	0.298

Comparison of the postoperative complications and VAS score for low-thermal plasma dissection device (PKC) and monopolar electrosurgery (CMES) groups.

*P -value is for testing the null hypothesis that population values (ranks sums or proportions) are equal between the two groups. Two-sided Fisher's exact test with a significance level of 5 % SD=standard deviation; VAS=visual analogue scale

**Fig. 1:**
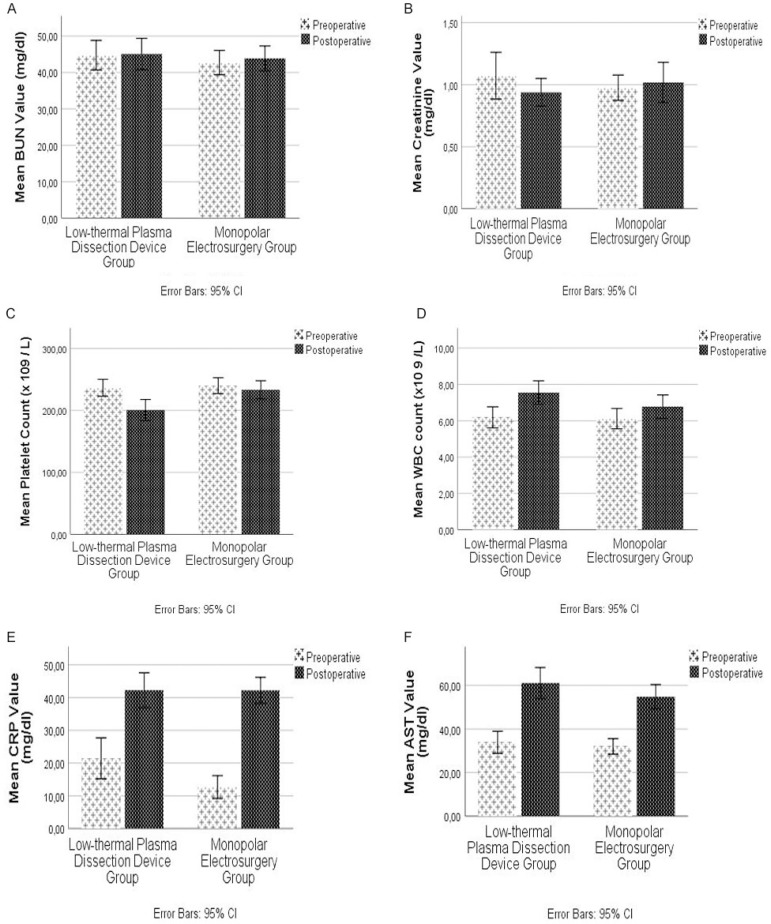
Comparison of the preoperative and postoperative hematologic and biochemical parameters between the low-thermal plasma dissection device (or plasma kinetic cautery [PKC]) group and the conventional monopolar electrosurgery (CMES) group. Statistical analysis of the differences between the groups was carried out with Mann-Whitney U test. The analyses of the preoperative and postoperative values of both groups were carried out with related-samples Wilcoxon signed-rank test. Two-sided P-value > 0.05 was considered statistically significant. A to D) Mean blood urea nitrogen (BUN) and creatinine value, and mean platelet and white blood cell (WBC) count were not found to be statistically significant in between-group analyses. Within-group analyses results are shown as related-samples Wilcoxon signed-rank test; 95% confidence interval (CI); lower, upper, P: CMES group BUN (-2, 3.5, P=0.645) and PKC group BUN (-4.5, 3, P=0.678), CMES group creatinine (0.08, 0.175, P<0.001) and PKC group creatinine (0.1, 0.17, P<0.001), CMES group platelet count (-18.5,19, P=0.98) and PKC group platelet count (-66,-26, P<0.001), and CMES group WBC (0.1, 1.15, P=0.02) and PKC group WBC (0.75, 2, P<0.001). E to F) The mean preoperative and postoperative C-reactive protein (CRP) (P=0.081 and P=0.539, respectively) and aspartate aminotransferase (AST) (P=0.960 and P=0165, respectively) values were not statistically significantly different in the between-group analyses. In within-group analyses, the postoperative values of both groups were significantly increased compared to preoperative values (CMES group CRP [26.45, 35.59, P<0.001] and PKC group CRP [13.98, 30.97, P<0.001], CMES group AST [15.28, 23.97, P<0.001] and PKC group AST [16.83, 29.51, P<0.001]).

Mean BUN, creatinine, platelet count, white blood cell count, CRP, and aspartate aminotransferase levels were increased compared to baseline levels in both groups, but this difference did not reach statistical significance (*P*>0.05 for all of the above parameters in two groups) (Figures 1 A to F).

**Fig. 2:**
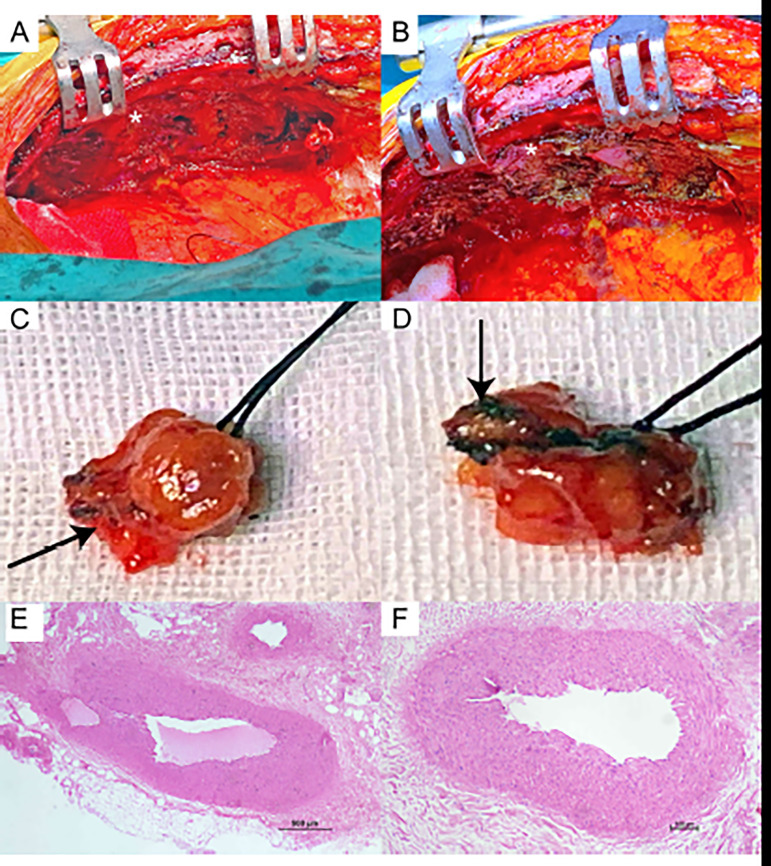
Intraoperative images. A) Image of the subcostal tissue after harvesting left internal mammary artery (LIMA) using the low-thermal plasma dissection device (or plasma kinetic cautery [PKC]) shows the presence of minimal thermal damage to the surrounding tissue (white asterisk). B) Image of the subcostal tissue after harvesting LIMA by the conventional monopolar electrosurgery (CMES) showing the presence of serious thermal damage to the surrounding tissue; burnt tissue can be clearly observed (white asterisk). C) Intraoperative image of excised thymus tissue by PKC incision shows a sharp cutting edge without any burning. D) Intraoperative image of excised thymus tissue by CMES incision shows an irregular cutting edge with significant burnt tissue. E to F) Histological features of LIMA graft after preparation with PKC (E) and CMES (F) at 500 µm (E) and 100 µm (F) represent similar findings with minimal effect.

The histopathological changes and the coagulation depths of the surgical procedure in the thymus tissue and LIMA specimens were obtained for qualitative purposes only and, therefore, a statistical comparison could not be carried out. Nonetheless, the depth of the tissue that was affected by coagulation was 1.52 mm with CMES and 1.29 mm with PKC. Endothelial damage of the LIMA was minimal with both devices (Figure 2).

## DISCUSSION

The purpose of this study was to evaluate if PKC could reduce bleeding complications and improve the wound-healing process in patients undergoing on-pump CABG. CABG entails a very large surgical incision site that includes median sternotomy and the entire course of MSV. The current study results show that PKC significantly reduced the incidence of bleeding-related reoperation, postoperative drain output, and time until drain tubes removal, surgical site pain, and need for blood transfusion. Abnormal wound healing was lower with PKC compared to the electrocautery technique, although the difference did not reach statistical significance and the wound healing process with PKC was better than the electrocautery technique from the perspective of the surgeon.

Although there are previously studies concerning PKC and CABG patients and data from surgeries with similar large surgical incision sites, such as abdominoplasty or mastectomy, have been reported, there are no studies comparing the use of PKC and CMES in post-CABG complications ^[3,10-12]^. To the best of our knowledge, this is the first study to report a comparison of the use of PKC and CMES in post-CABG complications.

In cardiac surgery, the large incision site requires the use of extensive cauterization and other instruments, such as vascular clips, sutures, and others, to reduce bleeding. Even with meticulous surgical technique, patients may have high amount of bleeding and drainage (hemorrhagic, serohemorrhagic, and serous, in due course, respectively) compared to other surgeries because of preoperative use of anticoagulant or antiplatelet therapy, intraoperative heparin administration, and postoperative hemodynamic distribution. Therefore, the best option is to manage coagulation concurrent with performing tissue incision. Although, CMES has been used safely during CABG for decades, PKC is a novel surgical instrument that provides minimal tissue injury, similar precision as a scalpel, and similar hemostatic control as CMES. Electrocautery generates high thermal energy, which can result in deep and wide coagulation of tissue and lysis of subcutaneous fatty tissue. These events are likely to be less with PKC, making PKC an attractive alternative. There are several studies showing better coagulation results in different surgery modalities. Dogan et al. ^[11]^ reported that PKC could reduce the drainage amount and duration compared to electrocautery in mastectomy patients. Ruidiaz et al. ^[10]^ reported that PKC generated 25.6% lower serous drain output compared to scalpel and traditional electrosurgery in abdominoplasty. Similar to these recent reports in the literature, the current study also demonstrated a statically significant decrease in drainage amount and time until drain tubes removal with PKC. The reduction in serous drainage was most likely due to successful vascular coagulation with minimal thermal injury with the use of PKC. Akgul et al. ^[4]^ reported that the depth of coagulation of the tissue from the cautery system is an important factor for the reduction of bleeding rate and successful hemostasis. These authors reported that the depth of coagulation with PKC was 3.2±1.53 mm and with monopolar electrocautery it was 1.52±1.29 mm in patients with benign prostate hyperplasia, which is a homogenous tissue. This is an important factor to consider during the preparation of both arterial and venous grafts and necessitates the preparation of grafts at a safe distance from cautery tip. Future randomized controlled studies on PKC with large sample sizes are needed to show the safety of PKC during graft preparation on CABG more clearly.

Blood transfusion due to postoperative bleeding in CABG leads to morbidities such as infection, prolongation of ventilation, neurological problems, and renal failure ^[1]^. Blood transfusion is carried out immediately in response to decreased hemoglobin level in CABG patients to prevent postoperative hemodynamic disturbances ^[1]^. The transfused erythrocyte suspension count is associated with postoperative drainage ^[12]^. If postoperative drainage is over the standard expectation and all options in the medical management of bleeding have been exhausted, emergency exploration for bleeding is mandatory in CABG. The current study results show that CMES patients required revision for bleeding surgeries significantly more than PKC patients. Ruidiaz et al. ^[10]^ reported a 58% reduction in hemoglobin drop with PKC compared to CMES in abdominoplasty patients. Although reduction in hemoglobin drop was not significant, erythrocyte suspension transfusion was significantly higher in the CMES group in the current study.

Our knowledge on the coagulation depth of LIMA and the surrounding tissue is limited. Since it is difficult to obtain these specimens from patients with ischemic heart disease during CABG *in vivo*, we aimed to compare this parameter on remnant thymus specimens that could be extracted during surgery in one case as a preliminary study. We cannot reach to any firm conclusion with just one sample; nonetheless, based on the surgeon’s experience and knowledge, the surgical site incisions with PKC appeared similar to the use of blade and worse pathological changes of the coagulated tissue were seen with CMES compared to PKC.

Postoperative pain is one of the most prevalent complaints of patients undergoing CABG during their hospital stay. Watt-Watson et al. ^[13]^ reported that 69% of patients had complaints of moderate to severe pain, which was severe for 30% of the patients at hospital discharge ^[14]^. Moreover, Parry et al. ^[14]^ reported that pain sensitivity increased from moderate to severe for deep breathing and coughing (43% of the patients), general activity (33% of the patients), mood (30% of the patients), and walking (27% of the patients). Generally, surgical incision-related wounds cause moderate to severe pain, which can be ameliorated over time, but can interfere with patients’ daily efforts such as cough, deep breath, or general activities ^[13-14]^.The current study showed a significant difference in pain intensity between the PKC and CMES groups, indicating the importance of surgical technique in the etiology of pain. Patients in the PKC group had a lower pain score compared to patients in the CMES group in the current study.

The high thermal energy of CMES results in widespread effects on the underlying tissue and lysis of subcutaneous fatty tissue leading to significantly abnormal wound healing. Ideal postsurgical healing is seen after scalpel incisions ^[10]^. A recent study stated that PKC shows healed incision strength and skin scar width equivalent to scalpel incisions ^[10]^. Recently, Huang et al. ^[15]^ investigated the coagulation depths of CMES and PKC in prostate resection in a canine model and stated that PKC resection caused a deeper coagulation depth in the prostatic tissue compared to CMES ^[15]^. According to these authors, their findings reflect a more efficient hemostatic effect of PKC. Corroborating this, we observed in the current study that the PKC group showed clinical signs of successful coagulation including reduced drainage volume and need for erythrocyte transfusion. However, in our opinion, this finding should be supported further with clinical or clinical simulation studies before advising clinicians on this difference in depth between PKC and CMES. Moreover, safety should be confirmed with histopathological examination of the arterial grafts especially during the preparation of LIMA grafts.

Although the current study has some limitations, it is important because it sheds light on the advantages of PKC over CMES.

### Limitations

There are limitations in this study that should be considered when interpreting the data. First, the patients received some anticoagulant and antiplatelet therapy prior to the surgery; however, it was impossible to discontinue these medications because of the presence of ischemic heart disease that resulted in bleeding and prolongation of the postoperative drainage. In our opinion, this was not a significant limitation of the study because every patient had the same clinical conditions. Second, although the differences in wound healing and complications did not reach statistical significance, based on the surgeon’s knowledge and experience, many differences were noted during the healing process of the wound. The current study method and statistical analysis were not designed to evaluate the healing process, but the observations made have initiated a new study plan to confirm the findings. Third, the data can be affected by the limited number of patients recruited from a single center. Fourth, the technique of the surgeon who carried out these surgeries may also have an effect on the study outcomes. Therefore, a multicenter randomized controlled study design with different surgeons and surgical techniques as well as large numbers of patients are needed to support our findings and in order to gain wider acceptance of the use of PKC in CABG.

## CONCLUSION

The low-thermal PKC appears to be effective and safe in performing dissection and hemostasis during CABG with its considerable reduced postoperative drainage, need for blood transfusion, reoperation, and postoperative pain. At the end of the procedure, PKC provides a better surgical outcome with minimal bleeding and reduced incidence of blood transfusion. The low thermal effect is a persuasive advantage of this technique for preparing grafts in a more reliable environment, especially for grafts as LIMA. The final conclusion of the study may be that PCK appears to be a good alternative to CMES for CABG.

**Table:** 

Authors' roles & responsibilities	
DU	Substantial contributions to the conception or design of the work; or the acquisition, analysis, or interpretation of data for the work; drafting the work or revising it critically for important intellectual content; agreement to be accountable for all aspects of the work in ensuring that questions related to the accuracy or integrity of any part of the work are appropriately investigated and resolved; final approval of the version to be published
EIİ	Final approval of the version to be published
